# Higher BMP Expression in Tendon Stem/Progenitor Cells Contributes to the Increased Heterotopic Ossification in Achilles Tendon With Aging

**DOI:** 10.3389/fcell.2020.570605

**Published:** 2020-09-25

**Authors:** Guangchun Dai, Yingjuan Li, Junyan Liu, Cheng Zhang, Minhao Chen, Panpan Lu, Yunfeng Rui

**Affiliations:** ^1^Department of Orthopaedics, Zhongda Hospital, School of Medicine, Southeast University, Nanjing, China; ^2^School of Medicine, Southeast University, Nanjing, China; ^3^Orthopaedic Trauma Institute (OTI), Southeast University, Nanjing, China; ^4^Trauma Center, Zhongda Hospital, Southeast University, Nanjing, China; ^5^Department of Geriatrics, Zhongda Hospital, School of Medicine, Southeast University, Nanjing, China; ^6^China Orthopedic Regenerative Medicine Group, Hangzhou, China

**Keywords:** aging, Achilles tendon, heterotopic ossification, tendon stem/progenitor cells, bone morphogenetic protein, osteogenic differentiation

## Abstract

Although the mineralization in tendon tissue has been reported in a series of aging and disease models, the underlying mechanisms remain unknown. This study aimed to describe the appearance of heterotopic ossification in rat Achilles tendon and further verify whether this tissue metaplasia is related to the enhanced osteogenic differentiation of tendon stem/progenitor cells (TSPCs) owing to the higher expression of bone morphogenetic proteins (BMP-2/4/7) with aging. The male SD rats, aged 4, 8, and 20 months (M), were used. The analyses of ossification and BMP expression in tendon were tested by radiological view (X-ray and CT), histological staining [hematoxylin and eosin (HE), Alcian blue, and Alizarin red], immunohistochemistry, and Western blot. The osteogenic differentiation potential and BMP expression of TSPCs were examined by Alizarin red S staining and real-time PCR. TSPCs were treated with BMP-2 or noggin, and the osteogenic differentiation potential was also examined. X-ray and CT showed the appearance of heterotopic ossification in tendon, and the volume and density of ossification was increased with aging. Histological staining showed the appearance of calcified region surrounded by chondrocyte-like cells and the increased osteogenesis-related gene and BMP expression in ossified tendon with aging. Moreover, the osteogenic differentiation potential and BMP expression in TSPCs isolated from ossified tendon were increased with aging. Additionally, BMP-2 increased the calcium nodule formation and osteogenesis-related gene expression in TSPCs. The addition of noggin inhibited BMP-induced enhancement of osteogenic differentiation. Thus, these findings suggested that the enhanced osteogenic differentiation of TSPCs contributes to the increased heterotopic ossification in aged tendon, which might be induced by the higher expression of BMPs with aging.

## Introduction

In our ever-aging population, a deep understanding of our musculoskeletal unit is of utmost importance. Age-related tendon disorders are one of the main causes for chronic pain, limited joint mobility, and tendon rapture among elderly patients. Heterotopic ossification has been reported in series models of injury and disease and plays an important role in uncommon conditions of tendon, including pathogenesis alterations (Rui et al., 2012b, 2013; Mao et al., 2019; Geng et al., 2020), which makes it as a vital target of tendon research. Moreover, a previous study has demonstrated that age-related tendon disorders is closely associated with the appearance of heterotopic ossification in tendon (Agabalyan et al., 2013). However, the underlying molecular mechanisms remain unclear and treatments are usually symptomatic.

Traditionally, tenocytes were considered the only cell present in tendon and played a critical role in tendon metabolism, repair, and regeneration. This hypothesis did not change until the isolation and identification of TSPCs in tendons, including mouse (Bi et al., 2007), human (Bi et al., 2007), rat (Rui et al., 2010), rabbit (Zhang and Wang, 2010), and fetal bovine (Yang et al., 2016). Although TSPCs represent a minor percentage of tendon cell compositions, these cells possess stem cell features such as self-renewal, clonogenicity, and multi-differentiation (Rui et al., 2010). After that, there was a substantial progress in the study of roles of TSPCs in tendon metabolism, repair, and regeneration. Compared with TSPCs isolated from healthy tendon, the proliferation of TSPCs isolated from collagenase-induced (CI) tendon injury model was significantly decreased, and the osteo-chondrogenic differentiation potential was increased, which might result in pathological ossification formation and failed tendon healing (Rui et al., 2013). Rui et al. (2011a) proposed that erroneous differentiation potential of TSPCs contributes to pathological alterations in calcified tendinopathy, which was consistent with the view of another study (Zhang X. et al., 2016). These findings supported the important role of altered TSPCs fate in pathological changes of chronic tendinopathy. During the aging process, TSPCs experienced an evident decrease in self-renewal and colony-forming ability and altered multi-differentiation capacity (Tan et al., 2012; Ruzzini et al., 2014; Dai et al., 2019; Li et al., 2019). TSPCs tended to differentiate into osteoblasts with over-passaging, which was a common cell senescence model *in vitro* (Tan et al., 2012). Ruzzini et al. (2014) indicated that aged TSPCs expressed higher levels of chondrogenic-related gene expression. Although there is no definite conclusion of these variations, the potential roles of altered TSPC differentiation capacity for age-related pathological changes in tendon were speculated (Dai et al., 2019; Li et al., 2019).

Ectopic expression of BMP-2/4/7 was detected in both clinical samples of tendinopathy (Rui et al., 2012b) and animal tendon injury model (Rui et al., 2013). The ectopic expression of BMPs was reported to facilitate non-tenogenic differentiation capacity of mesenchymal stem cells (MSCs) *in vitro*, including TSPCs (Lui, 2013; Zhang et al., 2019). BMPs might contribute to altered TSPCs’ fate through enhancing osteogenic differentiation and impairing tenogenic differentiation ability (Rui et al., 2012a; Lui and Wong, 2013). Moreover, ectopic expression of BMPs was also detected in subacromial bursa of patients with chronic degeneration of rotator cuff, which might induce the occurrence of ectopic bone/cartilage and accelerate structural degeneration of rotator cuff (Neuwirth et al., 2006). Taken together, these factors stimulated the current study to further describe the appearance and trend of heterotopic ossification in aged tendon and verify that this tissue metaplasia is related to the enhanced osteogenic differentiation potential of TSPCs, which is induced by the higher expression of BMPs. Moreover, BMPs might be an ideal therapeutic target for prevention or inhibition of heterotopic ossification formation in tendon age-related disorders.

## Materials and Methods

### Isolation and Culture of TSPCs

Animal experiments were approved by the Institutional Animal Care and Use Committee (IACUC) of the Southeast University School of Medicine. The procedures for TSPC isolation have been well established (Rui et al., 2010, 2011b). Briefly, the middle substance of Achilles tendon tissues per group was minced and digested with type I collagenase (3 mg/ml, Sigma-Aldrich, United States), and the cells were cultured in complete medium, which is low-glucose Dulbecco’s modified Eagle’s medium (LG-DMEM, Gibco, United States) containing 10% FBS, 100 U/ml penicillin, 100 mg/ml streptomycin, and 2 mM l-glutamine (Invitrogen, United States). TSPCs at passage 3 (P3) to P5 were used for all experiments. All experiments were repeated at least three times.

### Radiological Evaluation

Radiological evaluation was performed as previously described (Qiao et al., 2020). The analysis of heterotopic calcification was assessed by X-ray radiography and CT (computed tomography) scanning in 4M (young), 8M (middle-aged), and 20M (aged) male SD rats, and the aging model mainly refers to previous studies (Xing et al., 2016; Rui et al., 2019). Thirty-six rats were euthanized with CO_2_. X-ray radiography was performed with a Faxitron MX-20 X-ray machine at a voltage of 30 kV (Faxitron Bioptics, Wheeling, IL, United States). CT scans were acquired using a SkyScan 1176 radiograph microtomograph (BrukermicroCT, Kontich, Belgium) with an exposure time of 340 ms and a rotation step of 0.6°, a voltage of 65 kV, and a current of 385 μA. Reconstruction was performed with the NReconV.1.6.9.4 software using GPU acceleration. The ring artifact correction was fixed at 6, the smoothing was set at 1, the smoothing kernel was set at 2, and the beam hardening correction was set at 35%. After reconstruction, SkyScan CTAn (version: 1.13.8.1) was used for segmentation, registration, and quantification of all reconstructed images.

### General Histology and Immunohistochemistry Staining

The sections of Achilles tendons were washed in PBS, fixed in buffered formalin and 100% ethanol, embedded in paraffin, cut longitudinally to 5-mm thick sections, and mounted on 3-aminopropyl-triethoxy-silane (Sigma-Aldrich, United States)-coated slides. After deparaffinization, the sections were stained with H&E, Alcian blue, and Alizarin red staining, according to standard techniques. Briefly, the sections were reacted with Harris hematoxylin for 5 min and eosin for 3 min in H&E staining; the sections were reacted for 12 min in Alcian blue (pH 2.5) and for 8 min in Alizarin red staining solution (G1027 and G1038; Servicebio, China); the calcified region was stained light blue by Alcian blue, while the mineralization was stained red by Alizarin red. Images were captured using a digital camera and Q-Capture Pro software (ver. 6.0 Media Cybernetics, Bethesda, United States). Immunohistochemistry staining was performed as previously described (Rui et al., 2012b), and primary antibodies against BMP-2, OPN and OCN (Proteintech, China), and Runx2 (Abcam, United States) were used. Goat anti-rabbit (Chemicon, Temecula, CA, United States) or goat anti-mouse horseradish peroxidase (HRP)-conjugated secondary antibodies (Millipore, Billerica, MA, United States), together with 3,30 diaminobenzidine tetrahydrochloride (DAKO, Glostrup, Denmark), were used for signal detection. The sections from different age points were stained in the same batch and examined under light and polarized microscopies (DMRXA2 and DMRB, Leica Microsystems Wetzlar GmbH, Germany). The assessors were blinded to the grouping of the samples.

### Western Blot Assay

Western blot was performed to examine BMP-2/4/7 protein expression as previously described (Zhang J. et al., 2016). Achilles tendon from different age points was collected and protein was obtained from them. The protein was separated by 12% SDS-PAGE, transferred to PVDF membranes, and subsequently blocked in 5% fat-free milk for 2 h, followed by incubation with primary antibodies at 4°C overnight. All primary antibodies were from Bioworld Technology, Inc. Secondary antibody (Proteintech, China) conjugated with HRP was then applied. Finally, protein bands were detected with chemiluminescence (Beyotime, China). The protein expression levels assessed in this study was normalized to β-actin.

### Osteogenic Differentiation Assay

The multi-differentiation methods of TSPCs were elaborated in previous studies (Rui et al., 2010; Shi et al., 2019). TSPCs (P3) isolated from ossified tendon were seeded at 4 × 10^3^cells/cm^2^ in 12-well plates and cultured in complete medium until confluence. Afterward, they were cultured in complete basal or osteogenic induction medium (OIM), which was complete basal medium supplemented with 1 nM dexamethasone, 50 mM ascorbic acid, and 20 mM β-glycerolphosphate (Sigma-Aldrich, United States) for 21 days to assess mRNA expression of Runx2, OPN, OCN, BMP2, BMP-4, and BMP-7 by qRT-PCR and calcium nodule formation by Alizarin red S staining.

### Treatment of TSPCs With BMP-2 or Noggin

TSPCs isolated from ossified tendon aged 4M (abbreviated as Y-TSPC) and 8M and 20M (abbreviated as A-TSPC) were seeded at 4 × 10^3^ cells/cm^2^ in 12-well plates and cultured in complete medium until confluence. They were then cultured in OIM with or without recombinant human BMP-2 (rhBMP-2) (100 ng/ml; Solarbio, United States) (Rui et al., 2012a)/Noggin (0.25 μg/ml; Novoprotein, China) (Cho et al., 2005) for 7 days to assess mRNA expression of Runx2, OPN, and OCN by qRT-PCR and calcium nodule formation by Alizarin red S staining.

### Quantitative Real-Time PCR (qRT-PCR) Assay

TSPCs were seeded at 4 × 10^3^ cells/cm^2^ in six-well plates and cultured in complete medium or OIM until the desired time. Then, TSPCs were harvested and homogenized for RNA extraction with the Rneasy mini kit (Qiagen GmbH, Hilden, Germany). The mRNA was reverse transcribed to cDNA by the First-Strand cDNA kit (Promega, Madison, WI, United States). One microliter of total cDNA of each sample was amplified in the final volume of 20 μl of reaction mixture containing Power SYBR Green PCR Master Mix (Invitrogen Corporation, Carlsbad, CA, United States) and specific primers for target gene using the ABI StepOne Plus system (Applied Biosystems, CA, United States) (Supplementary Table S1). Cycling conditions were denaturation at 95°C for 10 min, 45 cycles at 95°C for 20 s, optimal annealing temperature (Supplementary Table S1) for 20 s, 72°C for 30 s, and finally at 60–95°C with a heating rate of 0.1°C/s. β-actin was used as an endogenous control. The data were calculated using the 2^–ΔΔ*CT*^ formula.

## Data Analysis

All data were presented as mean ± SD. Statistical analysis of the data was performed with SPSS (SPSS Inc, Chicago, IL, United States; version 22.0) using one-way ANOVA or unpaired Student’s *t*-test. *P* < 0.05 was regarded as statistically significant.

## Results

### Radiological Evaluation of Heterotopic Ossification in Achilles Tendon

**FIGURE 1:**
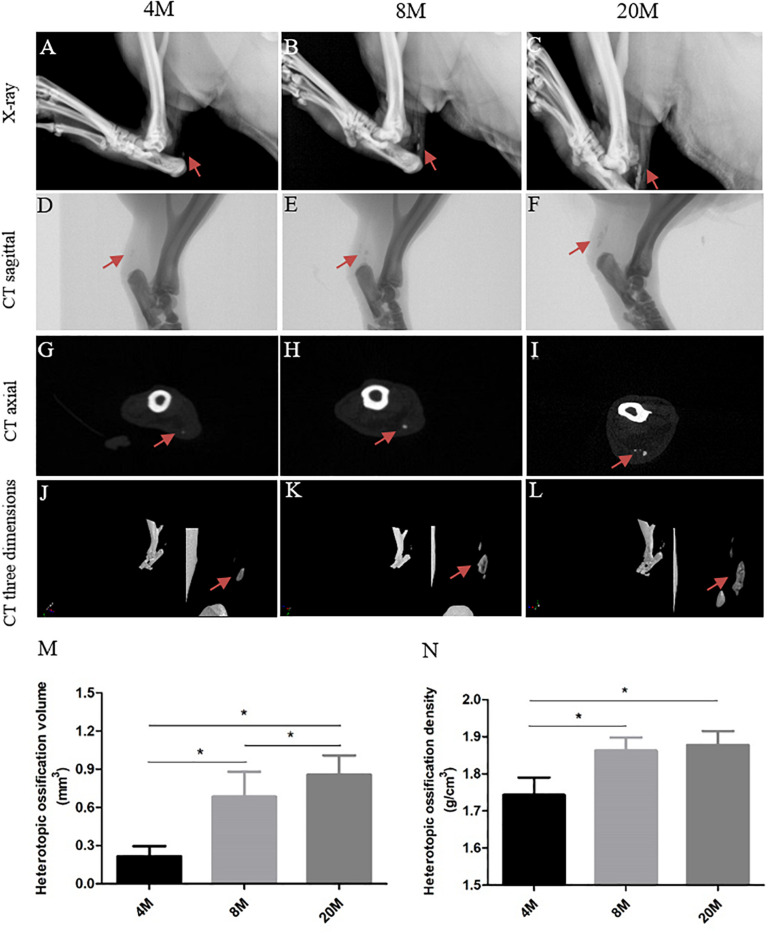
Imaging evaluation of heterotopic ossification. Imaging evaluation of heterotopic ossification in rat Achilles tendon aged 4, 8, and 20 M. **(A–C)** Representative X-ray figures from rats aged 4, 8, and 20 M. The visible radiopaque area presents the heterotopic ossification formation in tendon, and red arrows show the ossification region. Representative CT sagittal view **(D–F)**, CT axial view **(G–I)**, and CT three-dimensional view **(J–L)** figures from rats aged 4, 8, and 20M. Statistical diagrams show the increased trend with age in the volume **(M)** and density **(N)** of heterotopic ossification. ^∗^*P* < 0.05.

X-ray showed the formation of heterotopic ossification, which was presented by visible radiopaque area in tendon (red arrows), and the visible radiopaque area was increased in size with aging (Figures 1A–C). CT sagittal view (Figures 1D–F), CT axial view (Figures 1G–I), and CT three-dimensional view (Figures 1J–L) also observed the visible radiopaque area (red arrows). Moreover, we calculated the volume and density of heterotopic ossification, and there was an increased trend in the volume (Figure 1M) and density with aging (Figure 1N), but not from the density between group aged 8M and group aged 20M.

### Characterize Histological Alterations in Ossified Tendon With Aging

**FIGURE 2:**
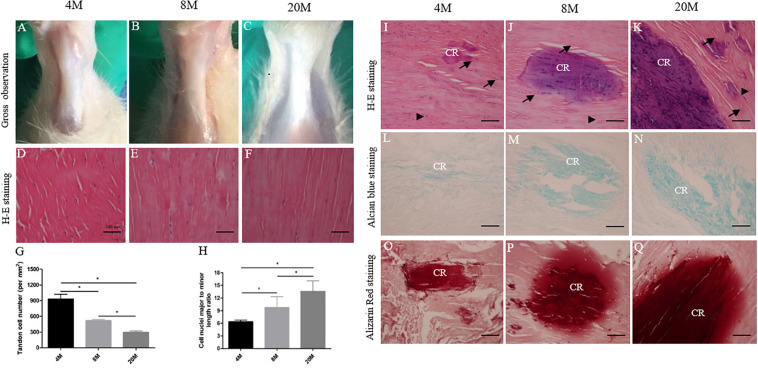
Histological alterations in ossified tendon with aging. Histology analysis. **(A–C)** Gross observation of Achilles tendon from rats aged 4, 8, and 20 M. **(D–F)** Representative H&E images of Achilles tendon from rats aged 4, 8, and 20 M mainly focused on tendon cells’ morphological appearance. Statistical diagrams show a decrease in cell number per mm^2^ **(G)** and a more flattened cell nuclei shape **(H)** in aged Achilles tendon. Representative H&E **(I–K)**, Alcian blue **(L–N)**, and Alizarin red **(O–Q)** staining images of ossified tendon from rats aged 4, 8, and 20 M; the heterotopic ossification was observed and the ossified area size was increased with age. Arrowhead: rounded tendon cells; arrow: chondrocyte-like cells; CR, calcified region. Scale bar = 100 μm. ^∗^*P* < 0.05.

Next, we performed histology staining to characterize alterations in ossified tendon with aging. It was observed that tendon appeared to have degenerative changes with water content of tissue gradually decreasing, structure becoming tight, and color tending to slight yellow on gross observation (Figures 2A–C) during the natural aging process. H&E staining displayed degenerative characteristics, including the appearance of spontaneous ectopic calcified region (CR) surrounded by chondrocyte-like phenotype cells (black arrows) (Figures 2I–K), a decrease of crimp morphology, a decrease in cell number per mm^2^ (Figures 2D–G), and a more flattened cell nuclei shape (Figures 2D–F,H) with aging. Alcian blue (Figures 2L–N) and Alizarin red staining (Figures 2O–Q) showed the appearance of spontaneous ossification region, the accumulation of cartilaginous matrix by Alcian blue staining and calcium nodules formation by Alizarin red staining, and the ossification region stained by Alcian blue and Alizarin red was increased in size with aging.

### The Osteogenesis-Related Genes Expression in Ossified Tendon Was Increased With Aging

**FIGURE 3:**
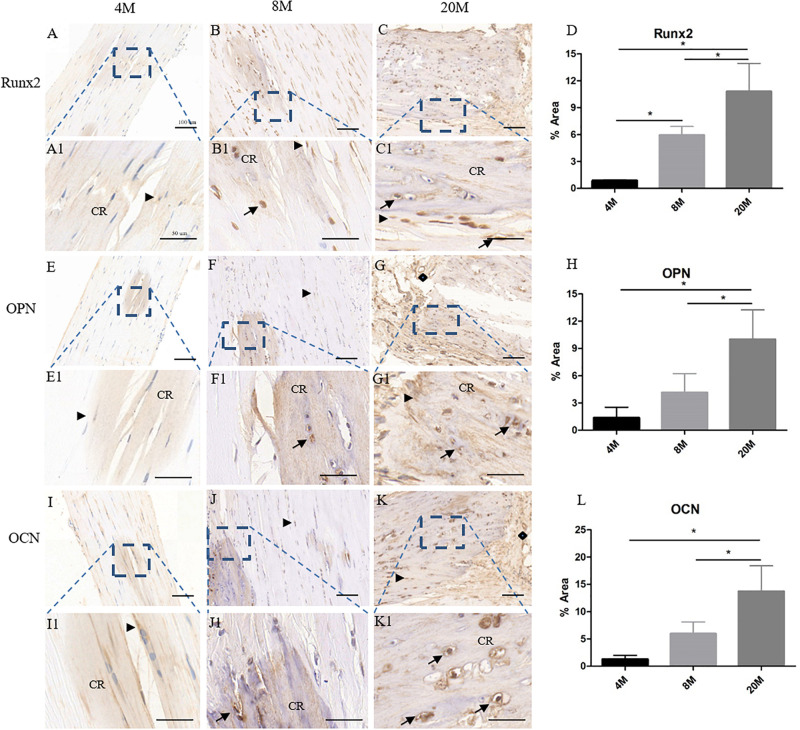
The osteogenesis-related genes expression in ossified tendon was increased with aging. Immunohistochemistry (IHC) staining of osteogenesis-related gene markers in ossified tendon aged 4, 8, and 20 M. IHC staining results revealed higher expression level of Runx2 **(A–C,A1–C1)**, OPN **(E–G,E1–G1)**, and OCN **(I–K,I1–K1)** in aged tendon. An increased trend with age of Runx2 **(D)**, OPN **(H)**, and OCN **(L)** expression was observed in ossified tendon by semi-quantitative IHC assays. Arrowhead: rounded tendon cells; arrow: chondrocyte-like cells; CR, calcified region; rhombus: blood vessels. Scale bar = 100 μm **(A–C,E–G,I–K)**; Scale bar = 50 μm **(A1–C1,E1–G1,I1–K1)**. ^∗^*P* < 0.05.

Additionally, immunohistochemistry staining of Runx2, OPN, and OCN was to evaluate osteogenesis-related gene expression levels in ossified tendon with aging. At the age of 4M, weak expression of Runx2 was observed in the rounded tendon cells (black arrowhead) and calcific region matrix, while the expression of Runx2 surrounding the calcific matrix was very weak to undetectable throughout the tendon (Figures 3A,A1). Weak expression of OPN (Figures 3E,E1) and OCN (Figures 3I,I1) was also observed mainly in the rounded tendon cells (black arrowhead) and the calcific region matrix of tendon from the group aged 4M. At the age of 8M, there was moderate expression of Runx2 in the rounded tendon cells (black arrowhead), chondrocyte-like cells (black arrow), and calcific region, and the expression of Runx2 in the tendon cells and their surrounding matrix around the calcific region was weak (Figures 3B,B1). Increased expression of OPN (Figures 3F,F1) and OCN (Figures 3J,J1) was also observed mainly in the rounded tendon cells (black arrowhead), chondrocyte-like cells (black arrow), and calcific region matrix of tendon from the group aged 8M, and the expression of OPN and OCN in the tendon cells and their surrounding matrix around the calcific region was weak. At the age of 20M, there was intense expression of Runx2 (Figures 3C,C1), OPN (Figures 3G,G1), and OCN (Figures 3K,K1) in the rounded tendon cells (black arrowhead), chondrocyte-like cells (black arrow), and calcific region matrix, and the expression of these genes in the tendon cells and their surrounding matrix was moderate throughout the tendon. As shown in Figures 3D,H,L, semi-quantitative results showed that the expression levels of Runx2, OPN, and OCN were significantly increased with aging, but not from the OPN and OCN expression between the group aged 4M and the group aged 8M. Overall, the expression level of osteogenesis-related genes was increased with aging.

### The Protein Expression of BMP-2/4/7 in Ossified Tendon Was Increased With Aging

**FIGURE 4:**
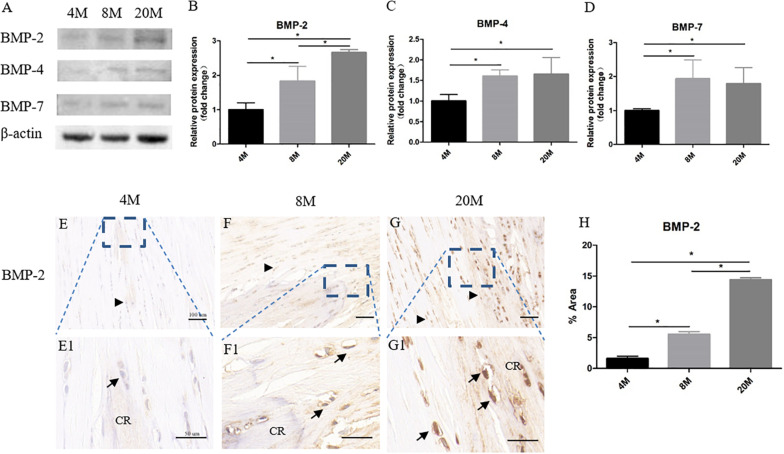
The protein expression of BMP-2/4/7 in ossified tendon was increased with aging. The protein expression of BMP-2/4/7 in ossified tendon aged 4, 8, and 20 M. **(A)** The protein of BMP-2/4/7 was detected by Western blotting. The semi-quantitative results revealed the protein expression levels of BMP-2 **(B)**, BMP-4 **(C)**, and BMP-7 **(D)** were increased with age. **(E–G,E1–G1)** IHC staining of BMP-2 in ossified tendon. The semi-quantitative results showed that the expression level of BMP-2 was increased with age **(H)**. CR, calcified region; arrowhead: rounded tendon cells; arrow: chondrocyte-like cells. Scale bar = 100 μm **(E–G)**; Scale bar = 50 μm **(E1–G1)**. ^∗^*P* < 0.05.

Western blot assay showed the protein expression of BMP-2/4/7 in ossified tendon was increased with aging (Figure 4A). Quantitative analysis of the intensity showed that there was an increased trend of BMP-2/4/7 protein expression in ossified tendon with aging, but not from the BMP-4/7 expression between group aged 8M and group aged 20M (Figures 4B–D). Moreover, the BMP-2 expression was also detected by immunohistochemical staining. At the age of 4M, weak expression of BMP-2 was detected in the rounded tendon cells (black arrowhead) and calcific region matrix, while the expression of BMP-2 surrounding calcific matrix was very weak to undetectable (Figures 4E,E1). At the age of 8M, there was a moderate expression of BMP-2 in the rounded tendon cells (black arrowhead), chondrocyte-like cells (black arrow), and calcific region, and the expression of BMP-2 in tendon cells and their surrounding matrix around the calcific region was weak (Figures 4F,F1). At month 20, there was intense expression of BMP-2 in the rounded tendon cells (black arrowhead), chondrocyte-like cells (black arrow), and calcific region matrix, and the expression of BMP-2 in tendon cells and their surrounding matrix was moderate throughout the tendon (Figures 4G,G1). Semi-quantitative result of the intensity showed that the expression of BMP-2 was increased with aging (Figure 4H).

### The Osteogenic Differentiation Potential of TSPCs Was Increased With Aging

**FIGURE 5:**
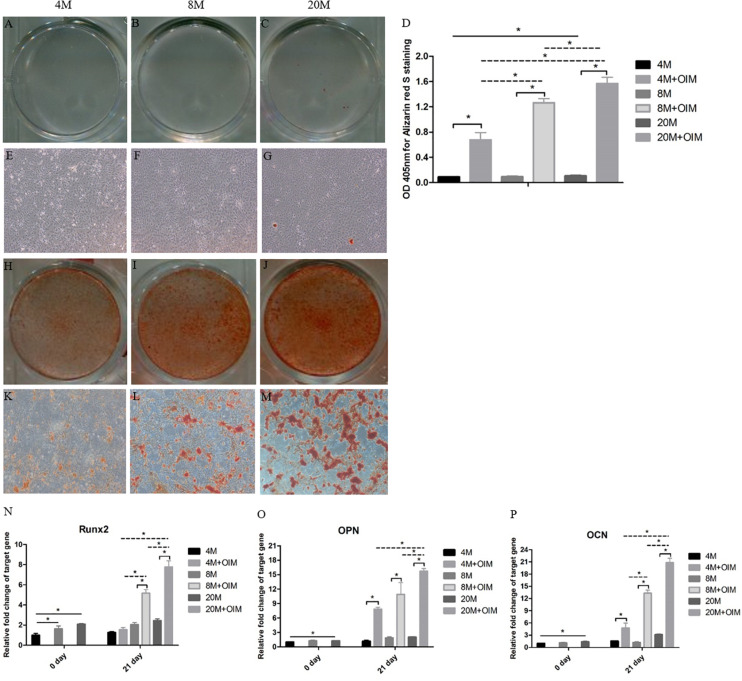
The osteogenic differentiation potential of TSPCs was increased with aging. The osteogenic differentiation potential of TSPCs isolated from ossified tendon aged 4, 8, and 20 M. Obvious calcium deposition was seen in osteogenic induction medium **(H–M)**, but not in basic medium **(A–C,E–G)**. **(D)** Statistical diagrams show the quantization of bound Alizarin red S in TSPCs. **(J)** Statistical diagram shows the expression levels of osteogenesis-related genes measured by qRT-PCR, including Runx2 **(N)**, OPN **(O)**, and OCN **(P)**. Magnification × 1 **(A–C,H–J)** and × 40 **(E–G,K–M)**. ^∗^*P* < 0.05.

At day 21, more alizarin red S (ARS)-positive calcium nodules were found in TSPCs cultured in OIM (Figures 5H–M) than basal medium (Figures 5A–G). Moreover, an increased trend of ARS-positive calcium of TSPCs with aging was observed in OIM (Figures 5H–M), and a slight increase of ARS-positive calcium of TSPCs with aging was observed in basal medium (Figures 5A–G). The quantification analysis showed that the TSPCs exhibited a significantly higher signal intensity of the calcium-bound ARS in induction medium than that in basal medium, and there was a significant increased trend with aging in induction medium (Figure 5D). In basal medium, there was an increased trend of signal intensity of the calcium-bound ARS between the group aged 4M and the group aged 20M (Figure 5D).

Moreover, the mRNA expression levels of Runx2, OPN, and OCN of TSPCs were remarkably upregulated after osteogenic induction compared with those in basal medium, but not the Runx2 expression of group aged 4M (Figures 5N–P). In OIM, the mRNA expression of Runx2, OPN, and OCN was significantly increased with aging apart from OPN expression between the group aged 4M and the group aged 8M (Figures 5N–P). At day 0, the expression of Runx2 was increased between the group aged 4M and the group aged 8M and between the group aged 4M and the group aged 20M, and the mRNA expression of OPN and OCN between the group aged 4M and the group aged 20M was also increased. Overall, these results showed that the osteogenic differentiation potential of TSPCs isolated from ossified tendon was upregulated with aging *in vitro*.

### The BMP-2/4/7 Expression of TSPCs Was Increased With Aging

**FIGURE 6:**
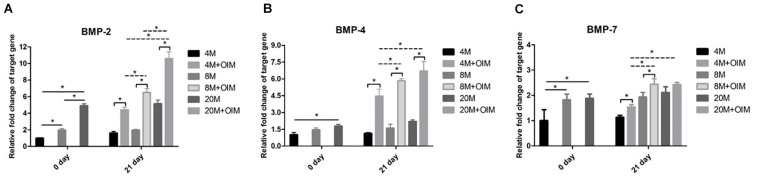
The BMP-2/4/7 expression of TSPCs was increased with aging. The BMP-2/4/7 expression of TSPCs isolated from ossified tendon aged 4, 8, and 20 M. Statistical diagrams show the expression of target genes measured by qRT-PCR, including BMP-2 **(A)**, BMP-4 **(B)**, and BMP-7 **(C)**. ^∗^*P* < 0.05.

At day 0, there was a weak mRNA expression of BMP-2 in TSPCs aged 4M, and an increased trend of BMP-2 expression with aging was observed (Figure 6A). The mRNA expression of BMP-2 was significantly upregulated after osteogenic induction compared with that in basal medium, and the expression of BMP-2 was increased with aging (Figure 6A). At day 0, there was an increased mRNA expression level of BMP-4 between the group aged 4M and the group aged 20M (Figure 6B). Upon the influence of induction medium, the mRNA expression of BMP-4 was upregulated compared with that in basal medium, and the expression of BMP-4 was increased with aging but not between the group aged 8M and the group aged 20M (Figure 6B). Meanwhile, the mRNA expression of BMP-7 was increased with aging but not between the group aged 8M and the group aged 20M at day 0 (Figure 6C). Upon the influence of induction medium, the mRNA expression of BMP-7 was significantly upregulated compared with that in basal medium but not in the group aged 20M, and the mRNA expression of BMP-7 was increased with aging but not between the group aged 8M and the group aged 20M (Figure 6C). Overall, the mRNA expression of BMP-2/4/7 of TSPCs isolated from ossified tendon was increased with aging *in vitro*.

### BMP-2 Stimulates the Osteogenic Differentiation Potential of Y-TSPCs

**FIGURE 7:**
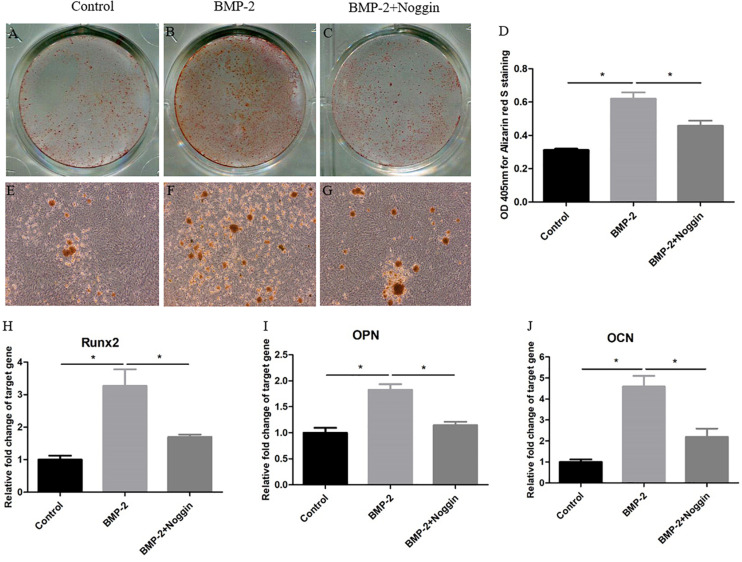
BMP-2 stimulates the osteogenic differentiation of Y-TSPCs. Effect of BMP-2 on the osteogenic differentiation of Y-TSPCs isolated from ossified tendon aged 4M. Graphs show that BMP-2 stimulates osteogenic differentiation tendency of TSPCs **(A,B,E,F)** and noggin inhibited BMP-2-induced potentiation of osteogenic differentiation **(B,C,F,G)** at day 7 as indicated by Alizarin red S staining. Statistical diagrams show the quantification of the amount of Alizarin red S bound to the calcified deposits in TSPCs with BMP-2 or noggin stimulation at day 7 **(D)**. Statistical diagrams show the mRNA expression of Runx2 **(H)**, OPN **(I)**, and OCN **(J)** in TSPCs upon treatment with BMP-2 or noggin in vitro. Magnification × 1 **(A–C)** and ×40 **(E–G)**. ^∗^*P* < 0.05.

To explore the effect of BMP-2 on the osteogenic differentiation of Y-TSPCs isolated from ossified tendon aged 4M, TSPCs were treated with BMP-2 or noggin (a BMP signaling inhibitor which binds to BMPs). At day 7, stimulation with BMP-2 increased the ARS-positive calcium nodules of TSPCs (Figures 7B,F) compared with that in the control group (Figures 7A,E), and the calcium-bound ARS signal intensity in the BMP-2-treated group was significantly higher than that in the control group (Figure 7D). Moreover, the mRNA expression of osteogenesis-related genes in TSPCs, including Runx2, OPN, and OCN, was significantly higher than those in the control group after treatment with BMP-2 at day 7 (Figures 7H–J). Stimulation with BMP-2 plus noggin reduced the ARS-positive calcium nodules (Figures 7C,G), calcium-bound ARS signal intensity (Figure 7D), and the expression of Runx2, OPN, and OCN (Figures 7H–J) in TSPCs compared with those in the BMP-2-treated group. Overall, BMP-2 stimulates the osteogenic differentiation potential of TSPCs *in vitro*, and the addition of noggin inhibited BMP-2-induced potentiation of osteogenic differentiation.

### Noggin Inhibits the Osteogenic Differentiation Potential of A-TSPCs

**FIGURE 8:**
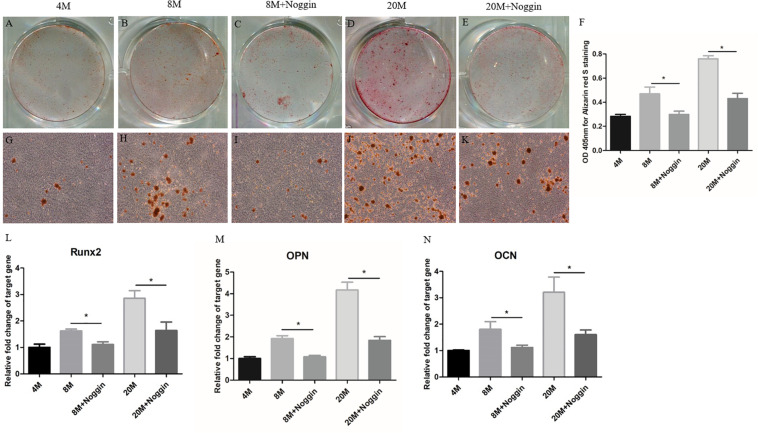
Noggin inhibits the osteogenic differentiation of A-TSPCs. Effect of noggin on the osteogenic differentiation of A-TSPCs isolated from ossified tendon aged 8 and 20M. Graphs show that noggin inhibits osteogenic differentiation tendency of TSPCs at day 7 **(A–E,G–K)** as indicated by Alizarin red S staining. Statistical diagrams show the quantification of the amount of Alizarin red S bound to the calcified deposits in TSPCs with or without noggin stimulation at day 7 **(F)**. Statistical diagrams show the mRNA expression of Runx2 **(L)**, OPN **(M)**, and OCN **(N)** in TSPCs upon treatment with noggin *in vitro*. Magnification × 1 **(A–E)** and ×40 **(G–K)**. ^∗^*P* < 0.05.

Then, noggin was used to inhibit the BMPs in A-TSPCs isolated from ossified tendon aged 8M and 20M. At day 7, Alizarin red S (ARS)-positive calcium nodules were found in TSPCs cultured in OIM (Figures 8A,G; B,H; and D,J), and stimulation with noggin reduced the ARS-positive calcium nodules of TSPCs aged 8M and 20M (Figures 8C,I and 8E,K) compared with that in the control group (Figures 8B,H and 8D,J), respectively, and the calcium-bound ARS signal intensity in the noggin-treated group was significantly lower than that in the control group (Figure 8F). Moreover, the mRNA expression of Runx2, OPN, and OCN in TSPCs was significantly lower than those in the control group after treatment with noggin at day 7 (Figures 8L–N), respectively. Overall, noggin inhibits the osteogenic differentiation potential of TSPCs aged 8M and 20M.

## Discussion

Our study firstly showed the appearance of heterotopic ossification in SD rat Achilles tendon and the increased trend of volume and density of ossification with aging. Moreover, the enhanced osteogenic differentiation potential of TSPCs isolated from ossified tendon might contribute to the heterotopic ossification, which is due to the higher expression of BMPs in TSPCs with aging.

Heterotopic ossification in tendon is a common clinical disease, often associated with tendon injury or surgery, or as a special manifestation of tendinopathy, and caused chronic pain and tendon rupture (Vaishya et al., 2019). In this study, we observed the appearance of heterotopic ossification in SD rat Achilles tendon, and the trend of volume and density of ossification was increased with aging. Heterotopic ossification of spinal ligament began in Tiptoe-walking mice at the age of 8 weeks, and the ossified area was increased with age (Liu et al., 2017). As shown in previous studies, heterotopic ossification is detrimental to normal healing of tendons and usually brings poor healing quality (Yee Lui et al., 2011; Rui et al., 2012b) and even accelerate other tissue aging progress (Pignolo et al., 2019). The healing ability of tendon tissues was decreased with aging (Tang et al., 2014; Dai et al., 2019), and heterotopic ossification is an important negative factor for tendon healing quality (Fang et al., 2014), indicating the occurrence of heterotopic ossification, and its increased trend in this study might cause the healing quality of aged tendon to weaken. Fang et al. (2014) showed that the choice of fetal fibroblasts as seed of tendon injury repair is better for the use of adult fibroblasts because of the decreased formation of heterotopic ossification, which always undermines tendon regeneration efficacy; meanwhile, the healing quality is improved. Thus, this study further demonstrates the hypothesis that heterotopic ossification and its increased tendency are harmful to the tendon healing process. Heterotopic ossification is normally thought as an important reason for the increased tendon stiffness and the risk of tendon rupture (Wood et al., 2011). According to the results of this study, the formation of heterotopic ossification is highest in the group aged 20M, and it should have the highest rate of tendon rupture. However, some clinical etiology observation reported that the middle-aged group has the highest incidence of tendon rupture, and the injury rate may also be related to the higher force placed on tendon (Kubo et al., 2007a,b). Thus, comprehensive factors may lead to the occurrence of tendon injury, and the different roles of these factors need to be further studied.

Currently, scholars have studied the causes for heterotopic calcification in tendon but have not formed a convincing theory (Agabalyan et al., 2013). Compared with embryonic avian tendon, heterotopic ossification within adult tendon seems to be the result of an endochondral process driven by its cells (Agabalyan et al., 2013). Previous studies showed that the non-tenogenic differentiation potential of TSPCs might result in the formation of heterotopic calcification in chronic tendon disease or following tendon injury (Agabalyan et al., 2013; Rui et al., 2013; Fang et al., 2014; Shi et al., 2019). In addition, Dai et al. (2019) and Li et al. (2019) proposed an intimate relationship between tendon aging and TSPC senescence, and the altered TSPC differentiation fate might play an essential role in the pathological changes of aged tendon. Based on these factors, we thus assess the altered trend of TSPC osteogenic differentiation potential with aging. In this study, the osteogenic differentiation potential of TSPCs was increased with aging, which is consistent with the increased trend of heterotopic ossification in aged tendon. A study showed that the osteogenic differentiation capacity of fetal fibroblasts was lower than that in adult fibroblasts, which result in less spontaneous ectopic ossification during tendon repair process (Fang et al., 2014). Meanwhile, spinal-ligament heterotopic ossification began in Tiptoe-walking mice aged 8 weeks, and the ossified area was increased with age, Alizarin red S assay also showed a higher osteogenic potential of MSCs isolated from Tiptoe-walking mice compared with that in wild-type mice (Liu et al., 2017). These findings further demonstrated that the higher osteogenic differentiation potential of stem cell might be responsible for the formation of tissue heterotopic ossification. Moreover, the abnormal differentiation potential of stem cells resident in other tissues was also considered to be causes for tissue ectopic calcification, such as vascular (Speer et al., 2009) and skeleton (Lounev et al., 2009). Taken together, it is reasonable that the increased osteogenic differentiation capacity of TSPCs might play an important role in the process of heterotopic ossification formation in tendon with aging.

The cytokines of BMP family, especially BMP-2/4/7, have been thought of as closely associated with tissue ectopic ossification formation and stem cell osteogenic differentiation potential. In the CI failed tendon healing animal model, chondrocyte phenotype and ossified region were observed (Lui et al., 2009), and the ectopic expression of BMP-2/4/7 was also observed around and in the chondrocyte-like cells and ossified region (Yee Lui et al., 2011), indicating the vital role of BMPs in the appearance of ectopic ossification, which was further corroborated by similar results observed in clinical specimens of tendinopathy (Rui et al., 2012b). In this study, the expression of BMP-2/4/7 in SD rat Achilles tendon was increased with aging, especially BMP-2, and its expression was consistent with the increased trend of heterotopic ossification and osteogenesis-related genes expression in tissue. Moreover, higher BMP-2 expression was observed around and in chondrocyte-like cells and ossified deposits, indicating that BMP-2 might account for the appearance of ectopic ossification in the overall process. BMP-2 was also responsible for the occurrence of ectopic ossification in the posterior longitudinal ligament (Harada et al., 2014), peripheral nerves (Olmsted-Davis et al., 2017), and rotator cuff (Neuwirth et al., 2006). In addition, stronger expression of BMP-2 and weaker expression of BMP-4/7 were observed in and around the chondrocyte-like cells and ossified region in ossified tendinopathy (Rui et al., 2012b), suggesting the possibility of BMP-2 playing a more vital role in this process. These results further confirmed the important role of BMP-2 in the occurrence of ectopic calcification in tendon, and BMP-2 might have been playing a key role throughout the process. However, another study showed that BMP-2 might not be involved in regulating the appearance of ossified area in the CI tendon injury model (Yee Lui et al., 2011); thus, more studies are needed to confirm the exact role of BMP-2. Moreover, BMP-4 also played a vital role in the heterotopic ossification formation after traumatic muscle injury (Kluk et al., 2012) and the idiopathic cutaneous ossification (Kim et al., 2008), and a similar role of BMP-7 in the formation of rotator cuff mineralization was observed (Neuwirth et al., 2006). Interestingly, in this study, the protein expression of BMP-4/7 in tendon between the group aged 8M and the group aged 20M was similar without significant difference as well as the gene expression of BMP-4/7 in TSPCs between the group aged 8M and the group aged 20M, indicating that BMP-4/7 might play an important role in the formation of ectopic ossification at the early stage and a weaker role at the late stage, which was corroborated by similar results of the key role of BMP-7 at the early phase of heterotopic ossification formation after muscle injury (Li et al., 2019). Based on these findings, BMP-2 might play a vital role in the occurrence of ectopic calcification in tendon throughout the process, while BMP-4/7 might play an indispensable role at the early stage. Different kinds of BMP might play their respective roles at different periods of the heterotopic ossification formation process, and the intricate underlying mechanism still needs further research. Previous studies also showed that treatment with BMP-2 promotes osteo-chondrogenic differentiation of TSPCs *in vitro*, and when TSPCs were disposed with BMP-2 and then transplanted subcutaneously into immunocompromised mice, structures similar to osteotendinous junctions were formed (Bi et al., 2007), which were similar to the ectopic ossified structures observed in both the tendinopathy animal model (Lui et al., 2009) and human samples (Rui et al., 2012b). In this study, the expression levels of BMPs in TSPC was increased with aging and upon osteogenic induction, indicating the intimate relationship between BMP expression and osteogenic differentiation potential of TSPC. In addition, BMP-2 could increase the osteogenic differentiation potential of TSPCs in this study, and BMPs also regulated differentiation ability of other cell species, such as tenocytes (Sato et al., 1988), embryonic stem cells (Gunne-Braden et al., 2020), and MSCs (Zhang et al., 2019). At the same time, we have tested the effect of noggin on the osteogenic differentiation of TSPCs *in vitro*, and the results showed that the addition of noggin could inhibit BMP-induced enhancement of osteogenic differentiation of TSPCs. These findings demonstrated that BMPs might increase the osteogenic differentiation potential of TSPCs, which is responsible for the increased trend of heterotopic ossification formation in tendon with aging. Meanwhile, the osteogenic differentiation potential was declined when the activities of BMP signaling are inhibited. In this study, we have verified the inhibitory effect of noggin on the osteogenic differentiation of TSPCs *in vitro*; moreover, implantation of noggin gene or muscle-derived stem cells over-expressing noggin were also reported to inhibit heterotopic ossification formation induced by BMP-4 in animal models (Glaser et al., 2003; Hannallah et al., 2004). In addition, other molecules, such as TGF-β neutralizing antibody (Wang et al., 2018) and transferrin receptor 2 (Rauner et al., 2019), and some drugs, such as insulin (Zhang et al., 2014) and desloratadine (Kusano et al., 2020), could attenuate heterotopic ossification formation induced by BMPs. A previous study also showed that mTOR signaling modulator suppressed heterotopic ossification formation (Hino et al., 2018); metformin exerted a dual negative regulatory effect on mTOR and BMP signal, and it might inhibit heterotopic ossification through an mTOR-BMP crosstalk signaling network (Wu et al., 2019). Recently, synthetic retinoid agonists, as a new option, has shown promise because it inhibited BMP-mediated heterotopic ossification formation in animal models (Sinha et al., 2016), and retinoid agonist therapy is being examined in patients with a rare, genetic form of heterotopic ossification (Lowery and Rosen, 2018). Thus, application of these factors might inhibit heterotopic ossification and promote tendon healing in tendinopathy and tendon injury models; further studies are needed to verify the effectiveness of these therapies for the management of heterotopic ossification formation during the tendon aging process. Although most of these strategies are at the stage of basic research, it is possible for them to enter clinical use when the distance between clinical medicine and basic research is resolved with more studies.

The limitation of this study is that the detailed signaling pathways of BMPs regulating the heterotopic ossification formation is not determined. Normally, BMP signaling mainly includes canonical SMAD signaling and non-SMAD signaling, and non-SMAD signaling is able to function by regulating SMAD signaling to form a crosstalk signaling network (Sanchez-Duffhues et al., 2015). A previous study showed that BMPs promote the osteogenic differentiation of TSPCs by phosphorylating SMAD in a failed tendon healing animal model (Lui and Wong, 2013), and SMAD was often a target of other cellular kinases, such as MAPK and GSK3-β, leading the crosstalk among these signals (Sanchez-Duffhues et al., 2015). Moreover, BMP-7 regulated the differentiation ability of MSC in the formation of heterotopic ossification of soft tissue through the Wnt/β-catenin pathway (Fu et al., 2017). In fibrodysplasia ossificans progressive disease, mTOR (Agarwal et al., 2016) and NF-κB/MAPK (Barruet et al., 2018) were related with the formation of muscle heterotopic ossification under crosstalk with BMP signaling, and BMPs together with SMAD, AKT, and mTOR/S6K signaling could prevent heterotopic ossification progress when the activity of PI3Kα was inhibited (Valer et al., 2019). RhoA-BMPs (Mu et al., 2013) and CGRP-SP-BMP-2 (Tuzmen et al., 2018) signaling also regulated heterotopic ossification progress in dystrophic muscle and Achilles tendon of mice, respectively. Additionally, BMP signaling pathway has crosstalk with Notch, FGF, and JAK/STAT signaling pathways in cell osteogenic differentiation potential and tissue ossification formation (Chen et al., 2012; Zhang et al., 2019, 2020). Thus, the factors from these signaling pathways might be involved in the formation of heterotopic ossification in aged tendon, and the detailed signaling pathways needed more studies. Moreover, Fang et al. (2014) have compared the form rate of heterotopic ossification with transplantation of mouse fetal or adult fibroblasts *in vivo*; thus, TSPCs isolated from different age points could be transplanted into nude mice to observe the formation of heterotopic ossification, which will further verify the hypothesis proposed in this study.

In summary, the appearance and the increased trend of heterotopic ossification were firstly reported in rat Achilles tendon with aging, and the enhanced osteogenic potential of TSPCs might contribute to the increased heterotopic ossification in aged tendon, which might be induced by the higher expression of BMPs in TSPCs with aging. These results might provide ideal prevention and treatment methods for heterotopic ossification in tendon, as well as other age-related tendon disorders.

## Data Availability Statement

All datasets generated for this study are included in the article/Supplementary Material.

## Ethics Statement

The animal study was reviewed and approved by Institutional Animal Care and Use Committee (IACUC) in Southeast University School of Medicine.

## Author Contributions

GD and YR conceived and designed the study. GD, YL, JL, and CZ performed the majority part of the experiments. MC and PL provided help for the experiments. GD, JL, and CZ acquired and analyzed the data. GD and YR drafted and edited the manuscript. All the authors aided in revising this manuscript for intellectual content and approved the final version to be published.

## Conflict of Interest

The authors declare that the research was conducted in the absence of any commercial or financial relationships that could be construed as a potential conflict of interest.
