# Supplementary material for: Higher BMP Expression in Tendon Stem/Progenitor Cells Contributes to the Increased Heterotopic Ossification in Achilles Tendon With Aging
Source: Front Cell Dev Biol. 2020 Sep 25;8:570605. doi: 10.3389/fcell.2020.570605 (PMC7546413; doi:10.3389/fcell.2020.570605)
Supplement: Supplementary file 1 [file Table_1.DOCX]

**Table S1** Primer Sequences and Condition for Real-Time Reverse Transcription-Polymerase Chain Reaction

| **Gene** | **Primer nucleotide sequence** | **Product**  **size (bp)** | **Annealing**  **temperature (8°C)** | **Accession no.** |
| --- | --- | --- | --- | --- |
| **β-actin** | **5’-ATCGTGGGCCGCCCTAGGCA-3’ (forward)**  **5’-TGGCCTTAGGGTTCAGAGGGG-3’ (reverse)** | **243** | **52** | **NM_031144** |
| **RUNX2** | **5’-CCGATGGGACCGTGGTT-3’ (forward)**  **5’-CAGCAGAGGCATTTCGTAGCT-3’ (reverse)** | **74** | **60** | **XM_346016** |
| **OPN** | **5’-TCCAAGGAGTATAAGCAGCGGGCCA-3’ (forward)**  **5’-CTCTTAGGGTCTAGGACTAGCTTCT-3’ (reverse)** | **200** | **55** | **AB001382.1** |
| **OCN** | **5’-GGTGCAAAGCCCAGCGACTCT-3’ (forward)**  **5’-GGAAGCCAATGTGGTCCGCTA-3’ (reverse)** | **199** | **60** | **M23637** |
| **BMP-2** | **5’-TAGTGACTTTTGGCCACGACG-3’ (forward)**  **5’-GCTTCCGCTGTTTGTGTTTG-3’ (reverse)** | **81** | **58** | **NM_017178** |
| **BMP-4** | **5’-CATGGCTCGCGCCTCCTAGC-3’ (forward)**  **5’-ATTCCGAGCGACGCACTGCC-3’ (reverse)** | **184** | **58** | **NM_012827** |
| **BMP-7** | **5’-CAACCTAGTGGAGCACGACAAGGA-3’ (forward)**  **5’-AGGTCGGACTCCCTGCCTGAGT-3’ (reverse)** | **213** | **60** | **NM_001191856** |
